# Muscle Contributions to Upper-Extremity Movement and Work From a Musculoskeletal Model of the Human Shoulder

**DOI:** 10.3389/fnbot.2019.00090

**Published:** 2019-11-05

**Authors:** Ajay Seth, Meilin Dong, Ricardo Matias, Scott Delp

**Affiliations:** ^1^Neuromuscular Biomechanics Lab, Bioengineering and Mechanical Engineering Departments, Stanford University, Stanford, CA, United States; ^2^Champalimaud Research and Clinical Centre, Champalimaud Centre for the Unknown, Lisbon, Portugal; ^3^Human Movement Analysis Lab, Escola Superior Saúde—Instituto Politécnico de Setúbal, Setúbal, Portugal

**Keywords:** computational shoulder model, scapula mechanics, thoracoscapular muscle work, serratus anterior, trapezius, deltoids, rotator-cuff muscles

## Abstract

Musculoskeletal models enable movement scientists to examine muscle function by computing the mechanical work done by muscles during motor tasks. To estimate muscle work accurately requires a model that is physiologically plausible. Previous models of the human shoulder have coupled scapula movement to humeral movement. While coupled movement produces a stereotypical scapulohumeral rhythm, it cannot model shrugging or independent movement of the scapula and humerus. The artificial coupling of humeral elevation to scapular rotation permits muscles that cross the glenohumeral joint, such as the rotator-cuff muscles and deltoids, to do implausible work to elevate and rotate the scapula. In reality, the motion of the scapula is controlled by thoracoscapular muscles, yet the roles of these muscles in shoulder function remains unclear. To elucidate the roles of the thoracoscapular muscles, we developed a shoulder model with an accurate scapulothoracic joint and includes scapular muscles to drive its motion. We used the model to compute the work done by the thoracoscapular muscles during shrugging and arm elevation. We found that the bulk of the work done in upper-extremity tasks is performed by the largest muscles of the shoulder: trapezius, deltoids, pectoralis major, and serratus-anterior. Trapezius and serratus anterior prove to be important synergists in performing upward-rotation of the scapula. We show that the large thoracoscapular muscles do more work than glenohumeral muscles during arm-elevation tasks. The model, experimental data and simulation results are freely available on SimTK.org to enable anyone to explore our results and to perform further studies in OpenSim 4.0.

## Introduction

Abnormal scapular movement is indicative of shoulder dysfunction, such as subacromial impingement, rotator-cuff tears, and other injuries (Struyf et al., 2011). A symptom of shoulder dysfunction is scapular dyskinesia (Kibler et al., 2013), including scapular winging (Martin and Fish, 2008), in which the medial border of the scapula lifts off the thoracic surface. Before researchers can investigate shoulder dysfunctions, we require biomechanical models with the degrees of freedom and musculature attached to the scapula, which is currently unavailable.

Models designed to understand glenohumeral injury and rehabilitation (Garner and Pandy, 2001; Holzbaur et al., 2005; Dickerson et al., 2007; Chadwick et al., 2009; Bolsterlee et al., 2013; Saul et al., 2015) ignore muscle actions of the largest thoracoscapular muscles: trapezius, rhomboids, and serratus-anterior (Rockwood, 2009). These muscles likely play important roles in human upper-extremity movements given their size and force-generation capacity. While Odle et al. (2019) included the rhomboids and serratus-anterior muscles in their model, they maintained the scapulohumeral coupling from the model reported by Saul et al. (2015), which does not need thoracoscapular muscles to move. We can only assume that coupling scapular kinematics to humeral rotation yields the perplexing results that the rotator-cuff muscles generate the largest forces during the recovery phase of wheel-chair propulsion, while the larger superior trapezius, rhomboids, anterior deltoid, and pectoralis major muscles produced virtually no force throughout the movement (Odle et al., 2019).

The model by van der Helm (1994a), was the first to include thoracoscapular muscles and enable realistic scapula kinematics by including scapular contact with the thoracic surface. While numerous models (van der Helm, 1994b; Garner and Pandy, 2001; Dickerson et al., 2007; Dubowsky et al., 2008; Odle et al., 2019) have computed thoracoscapular muscle forces for a variety of upper-extremity tasks, the work performed by these muscles during these tasks was not reported.

We have developed a musculoskeletal model of the shoulder that includes the large thoracoscapular muscles and the kinematically uncoupled movement of the scapula so that we may answer two fundamental questions about upper-extremity muscle function. First, how much work is done by the thoracoscapular and glenohumeral muscles during shoulder shrugging and arm-elevation tasks? Second, what motions of the scapula are controlled by large thoracoscapular muscles such as trapezius and serratus anterior during these shoulder tasks?

## Methods

### Model of the Human Shoulder

**Figure 1:**
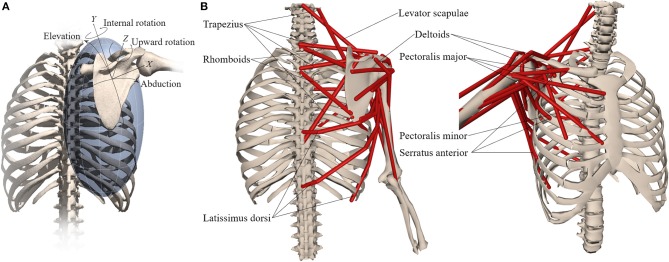
Musculoskeletal model with **(A)** scapula degrees-of-freedom and **(B)** shoulder muscles that control the scapula.

We developed a model of the human shoulder in OpenSim (Delp et al., 2007; Seth et al., 2018) (Figure 1) that combines a fast and accurate skeletal model of scapulothoracic kinematics (Seth et al., 2016) with muscle paths and architecture based on (Klein Breteler et al., 1999). To reduce complexity and improve computational performance of the model, muscle bundles from van der Helm (1994a) were aggregated and their parameters combined (Table 1). Muscle paths including wrapping surfaces and their geometry were adjusted to produce moment arms bounded by measurements from cadaver experiments (Ackland et al., 2008). Continuity of muscle moment arms were verified over the full range-of-motion of the model.

**Table 1:** Thoracoscapular shoulder model muscle parameters adapted from Klein Breteler et al. (1999) with aggregated bundles from by van der Helm (1994a).

**Muscle**	**Group**	**Max isometric force**	**Optimal fiber length**	**Tendon slack length**	**Pennation Angle**	**van der Helm bundles**
Trapezius	Scapula superior	1043	0.1127	0.027	0	1–6
	Scapula middle	470.4	0.0832	0.032	0	7–9
	Scapula inferior	414.4	0.1264	0.035	0	10-12
	Clavicle	201.6	0.1116	0.027	0	C1-C2
Serratus anterior	Superior	387.8	0.0945	0.000	0	9-12
	Middle	508	0.1538	0.012	0	5-8
	Inferior	430	0.1587	0.000	0	1–4
Rhomboideus	Superior	200.2	0.0986	0.015	0	1–2
	Inferior	407.4	0.1152	0.028	0	3–4
Levator scapulae		280	0.1578	0.019	0	All
Coracobrachialis		648.2	0.0683	0.104	0	All
Deltoideus	Anterior	707.7	0.0940	0.088	5	C1–C4
	Middle	2597.8	0.0748	0.064	5	4–11
	Posterior	1324.4	0.0949	0.076	5	1–3
Latissimus Dorsi	Superior	201.6	0.2109	0.081	0	1–2
	Middle	315	0.2656	0.095	0	3–4
	Inferior	270.2	0.3062	0.062	0	5–6
Pectoralis Major	Clavicle	408.8	0.1087	0.014	0	C1–C2
	Thorax middle	683.2	0.1500	0.026	0	4–6
	Thorax inferior	571.2	0.1830	0.043	0	1–3
Teres Major		851.2	0.1410	0.006	0	All
Infraspinatus	Superior	967.4	0.0698	0.050	0	4–6
	Inferior	1037.4	0.0677	0.084	0	1–3
Pectoralis minor		429.8	0.1183	0.032	0	All
Teres minor		695.8	0.0550	0.051	0	All
Subscapularis	Superior	540.4	0.0676	0.059	5	1–3
	Middle	609	0.0744	0.055	5	4–5,10
	Inferior	854	0.0721	0.059	0	6–9, 11
Supraspinatus	Anterior	543.2	0.0554	0.031	0	3–4
	Posterior	326.2	0.0591	0.025	0	1–2
Triceps long		1580.6	0.0969	0.241	10	All
Biceps	Long	485.8	0.1412	0.257	0	All
	Brevis	693	0.1264	0.212	0	All

Model scaling and inverse kinematics were performed in OpenSim to compute model joint angles from experimental marker data (see below). Bones and corresponding joint locations and muscle attachment locations were scaled linearly based on marker-based distances between the subject and the base (generic) model. Muscle optimal fiber and tendon slack lengths were scaled to preserve their ratio over the muscle path length in the scaled model. The ellipsoid surface of the thorax in the scapulothoracic joint was scaled by optimizing the ellipsoid tilt and radii that minimized marker-tracking errors. The thorax muscle wrapping object was initially scaled according to the thorax scale factors, however this lead to serratus anterior insertions on the anterior scapula to enter the wrapping surface, which results in the wrapping path becoming undefined. The wrapping ellipsoid surface was then hand adjusted by tilting the top of the ellipsoid toward the sternum until the path of the serratus anterior was well-defined for the complete scapula range of motion across all tasks. The greater freedom of the scapula also resulted in some muscles exceeding 150% of the optimal fiber-length and/or being too short (<50%) resulting in their inability to produce active force during the range-of-motion of the anticipated tasks. In these situations, the muscle optimal fiber-length was incrementally increased (by 2%) and tendon slack-length reduced by the same length until muscle forces alone were sufficient to track desired task kinematics. See Table 1 for the complete set of muscle parameters implemented in the shoulder model.

Computed muscle control (CMC) (Thelen et al., 2003) was used to generate muscle-driven simulations that tracked joint angles from inverse kinematics. All simulations were performed using OpenSim 4.0 (Seth et al., 2018) on a desktop computer with an Intel i7 3930K 3.2GHz processor and 32GB of RAM. All computations were evaluated running on a single CPU core.

### Experimental Data Collection and Comparison Methods

To test the shoulder model, we collected upper-extremity kinematics using Ascension 3D trakSTAR (Ascension Technology Corp, USA) and Motion Monitor software (Innovative Sports Training, Chicago, Illinois) to simultaneously and continuously track four miniaturize sensors (model 800) at a sampling rate of 120 Hz. Three sensors were fixed to the thorax, scapula and humerus, respectively. Prior to continuous collection, a fourth sensor was rigidly affixed to a stylus and used to digitize the locations of bony landmarks with respect to the corresponding sensors, while the subject was in a neutral pose. The thorax sensor was placed on the T1 spinous process; the scapula sensor was placed over the flat surface on the superior acromion. Both sensors were held in place with double-sided adhesive tape wrapped with EnduraSports tape (Endura-Tape). The arm sensor was fixed on a strap that was tightly adjusted around the lateral aspect of the most distal part of the humerus. The ISB shoulder protocol (Wu et al., 2005) implemented in the MotionMonitor software was used to collect data based on the recorded sensor and digitized landmark locations (Ludewig et al., 2009) and identified as markers in OpenSim.

Surface electromyography (EMG) electrodes were placed on the skin after preparation (Basmajian and de Luca, 1985) according to Cram (2010) with an interelectrode distance of 20 mm over the: superior, middle and inferior trapezius; serratus anterior; anterior, middle, and posterior deltoids; infraspinatus; teres major; pectoralis major (clavicular), and latissimus dorsi muscles. A reference electrode was placed on the contralateral acromion. We collected three trials of shoulder shrugging, forward flexion and abduction without and with a 2 kg hand-held weight, for a total of 18 trials from the dominant shoulder (right) of a 26-year-old healthy female subject (height: 162 cm, weight: 52 kg). The experimental protocol was approved by the ethics committee of the Polytechnic Institute of Setúbal.

We processed the raw EMG by high pass filtering at 100 Hz, full-wave rectifying the resultant signal, and then low-pass filtering at 4 Hz to obtain EMG envelopes according to ISEK (Merletti, 1999). Processed EMG envelopes were normalized by maximum voluntary contractions obtained according to (Kendall et al., 2005).

We compared muscle computed activations to processed EMG waveforms by computing the mean-absolute error (MAE) over the shoulder task interval (de Zee et al., 2007; Dubowsky et al., 2008; Odle et al., 2019) for each muscle across all tasks. For serratus anterior, the average activation of the three muscle bundles in the model was used in the comparison.

To understand the contribution of individual muscles to shoulder movement in our subject, we calculated the work done by muscles by integrating the positive muscle power during scapular and humeral elevation. Muscle power was computed from the product of muscle-tendon unit force (from CMC) and shortening velocity, where concentric contractions yield positive power. The total positive muscle work during the elevation phase of the tasks was compared to the external work computed as the change in model potential energy due to elevating the arm (and added mass) against gravity. We expected the positive muscle work to be greater than external work due to negative work of lengthening muscles and the acceleration of limb segments relative to the center-of-mass.

## Results

We generated muscle-driven simulations for all (18) experimental trials. Inverse kinematics accuracy for each trial was within 1 cm RMSE with respect to experimental marker locations and computed within 1.3 × of real-time. The average computation to real-time ratio for all CMC muscle-driven simulations was below 400 compute/real time. Table 2, presents the compute to real-time ratio for simulating our model for each task. For comparison, we obtained a 4–17 × speedup when executing CMC with our model vs. the model by (Saul et al., 2015) for the flexion and abduction tasks.

**Table 2:** Model computation vs. real time ratio (compute/real) by task.

**Task**	**IK**	**CMC**	**FD**
Shrug	1.3	377	11
Shrug+	1.3	404	18
Flexion	1.2	408	13
Flexion+	1.1	401	18
Abduction	1.0	384	17
Abduction+	0.9	385	17

*Lower values are faster. Computation times evaluated for inverse kinematics (IK), computed muscle control (CMC) and forward dynamic (FD) simulations*.

*+Indicates the task with a 2 kg hand-held mass*.

**Figure 2:**
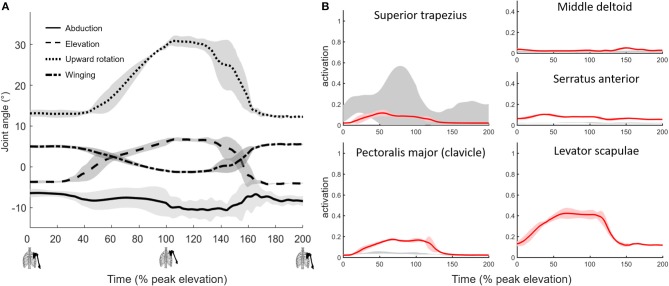
Muscle-driven simulation of shoulder shrugging. **(A)** Scapulothoracic joint kinematics and **(B)** simulated muscle activations (red, bold mean ± 1 SD shaded) compared to EMG (±1 SD gray shaded).

Muscle activations from muscle-driven simulations of the shoulder model were compared to the EMG for the same tasks, which yielded an average MAE of 0.06, with the vast majority of measured muscles below 0.1 (Table 3). The Pectoralis major muscle showed the worst agreement during the shrugging task (without a handheld weight) where EMG was relatively silent in the depression phase, while the model estimated low but consistent activation throughout the movement (Figure 2B).

**Table 3:** Mean absolute error between subject EMG and model muscle activations across tasks.

**Task**	**Superior trapezius**	**Middle trapezius**	**Inferior trapezius**	**Serratus anterior**	**Anterior deltoid**	**Middle deltoid**	**Posterior deltoid**	**Infra-spinatus**	**Teres Major**	**Pec.Maj. clavicle**	**Latissimus dorsi**
Shrug	0.09	0.01	0.01	0.05	0.01	0.01	0.01	0.05	0.01	0.06	0.02
Shrug+	0.05	0.02	0.01	0.08	0.01	0.05	0.02	0.01	0.02	0.01	0.08
Flexion	0.05	0.04	0.03	0.05	0.04	0.06	0.02	0.02	0.05	0.04	0.08
Flexion+	0.07	0.05	0.03	0.08	0.05	0.07	0.05	0.03	0.08	0.05	**0.15**
Abduction	0.10	0.07	0.05	0.05	0.09	0.08	0.06	0.04	0.02	0.01	0.04
Abduction+	0.10	0.05	0.04	0.08	0.05	0.07	0.08	0.08	0.04	0.02	**0.11**

*A value below 0.1 corresponds to <10% difference between two signals. Values > 0.1 are in bold. +Indicates the task with a 2kg hand-held mass*.

The simulated shoulder shrug demonstrates that the model can elevate and rotate the scapula independent of humerus rotations (Figure 2A). Simulated muscle activity during shrugging indicates that levator scapulae elevates the scapula while superior trapezius may both elevate and upward rotate the scapula during shrugging (Figure 2B).

**Figure 3:**
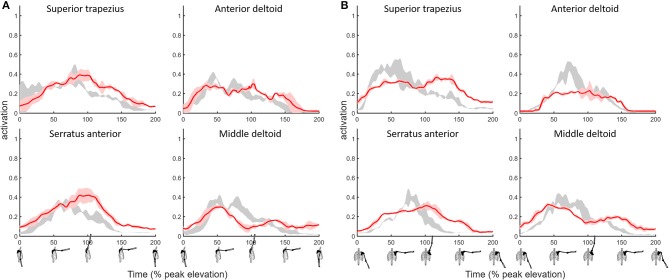
Shoulder model muscle activations for primary muscles used to elevate the humerus during the **(A)** flexion and **(B)** abduction tasks with a 2 kg hand-held mass. Simulated muscle activations (red, ±1 SD shaded) compared to EMG (gray shaded).

The MAE values for superior trapezius, deltoids and serratus anterior muscle activations when compared to EMG during shoulder flexion and abduction tasks (Figure 3) where 0.1 or below (Table 2) indicative of a high quantitative correlation (Morrow et al., 2010; Odle et al., 2019) between simulated and subject muscle activity.

**Figure 4:**
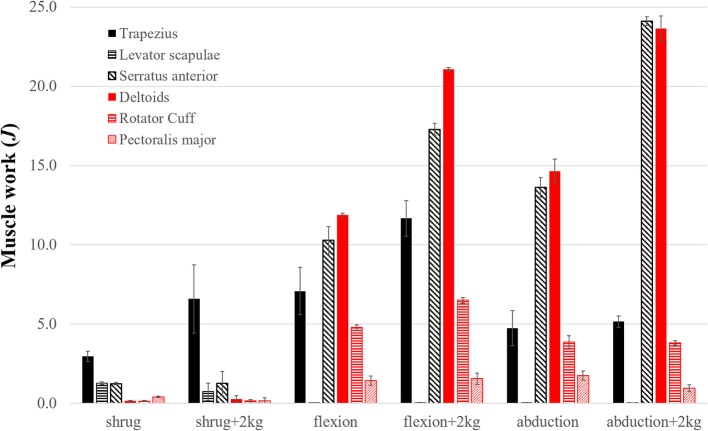
The positive work (*J*) done by the top contributing shoulder muscles during the elevation phase of each task. Shaded bars are the work averaged over three trials and thin error bars are ± SD. Top seven contributors are grouped into thoracoscapular (black) and glenohumeral (red) muscles. Excluded muscles performed <3% of the total muscle work.

Superior trapezius, serratus anterior, and deltoids showed the greatest muscle activity and did the most positive work during the elevation phase of each task (Figure 4). As expected, the total positive muscle work was consistently greater than the external work. For example, the total positive muscle work of 61.6*J* exceeded the total external work (49.5*J*) necessary to elevate the arm during abduction with a 2 kg handheld weight.

## Discussion

We have developed a musculoskeletal shoulder model that reproduces the observed skeletal kinematics and muscle activity during shoulder shrugging and arm-elevation tasks. The model enabled us to compute the work done by upper-extremity muscles that drive the scapula and the glenohumeral joint. Prior to this study, scapulothoracic interaction was modeled either by forces of deformation using finite elements (van der Helm, 1994a) or by contact point constraints (Garner and Pandy, 1999) making use of these models challenging. The inherent model stiffness due to large (muscle and scapulothoracic contact) forces and a low mass scapula body has required custom system dynamics and contact formulations and the use of implicit integration (Chadwick et al., 2014) that are not widely accessible to the clinical and rehabilitation communities. Available models that couple humeral elevation to scapular rotation (Saul et al., 2015; Odle et al., 2019) are unable to accurately account for the muscle work required to move the scapula and the subsequent upper-extremity. We show that a model can capture scapular kinematics and account for muscles that drive the scapula, without a detriment to computational performance. In fact, the model computes 4–17 × faster than a comparable model (Saul et al., 2015) without these capabilities while allowing researchers to study the function of thoracoscapular muscles.

We simulated shrugging, flexion, and abduction tasks with/out a 2 kg hand held weight using our shoulder model. We found agreement between the simulated model and subject measurements with marker tracking within 1 cm RMSE, and model activation compared to subject EMG with an average MAE below 0.1 for the most active muscles during the tasks we examined. While we did not directly measure muscle forces or velocities, the agreement of model kinematics and muscle activity give us confidence that the muscle work computed by the model is representative of the relative work done by the subject's shoulder muscles. One of the main benefits of complimenting experimental measures with a computational model, is that we can estimate quantities that are difficult to measure such as muscle force and work.

Our first aim was to evaluate how much work is done by the thoracoscapular and glenohumeral muscles during shoulder shrugging and arm-elevation tasks? To address this aim, we computed the work done by individual shoulder muscles during the simulated shrugging, flexion and abduction tasks (Figure 4) using the shoulder model. We found (superior) trapezius, serratus anterior, and rhomboids (i.e., the large thoracoscapular muscles) combined to exceed the work of the deltoids, rotator-cuff, and teres major (i.e., the glenohumeral muscles). While deltoids were the largest muscle contributor to humeral elevation during flexion tasks, trapezius and serratus anterior combined to do more work than deltoids for every task including flexion.

Our second aim was to answer what motions of the scapula are controlled by large thoracoscapular muscles such as trapezius and serratus anterior during these shoulder tasks? We addressed this question by analyzing which thoracoscapular muscles perform work on the scapula during shoulder tasks. Our results show that levator scapulae elevates the scapula while trapezius and serratus anterior upward rotate the scapula during shrugging. As work demands increase due to a handheld weight, we found that superior trapezius and serratus anterior work together to form a powerful force-couple to upward-rotate the scapula during arm-elevation tasks. These results confirm the function of superior trapezius and serratus anterior muscles as described by anatomy textbooks (Stranding, 2016).

The implications of these results for human rehabilitation and neurorehabilitative robotics is significant. Examining the functional roles of the major upper-extremity muscles is key to understanding which muscles to assist and when to apply assistance.

The shoulder model provides unique opportunities to design and test rehabilitative strategies directly in a physics and physiologically consistent way. In the same way that simulation was used to test ideal-assistance in human running (Uchida et al., 2016) it can be applied to explore upper-extremity assistance strategies that enable a weakened model to reach target locations that minimizes device weight and power use. We can use the model to discover principles for upper-extremity assistance that enable patients to perform independently and effectively.

In patients with shoulder pathologies, for example due to brachial nerve palsy, the model enables us to test hypotheses about the causes and cures for scapula dyskinesia. There is mounting evidence that altered scapula kinematics is indicative of shoulder pathologies (Ludewig and Reynolds, 2009; Kibler et al., 2013), and scapula-focused treatments improve outcomes in patients with shoulder disorders (Struyf et al., 2013; Hotta et al., 2018). The biomechanics underlying these improvements, however, are poorly understood. Therefore, clinicians require both reliable measurements and accurate models to examine how muscles cause both healthy and pathological movements. We have shown that the thoracoscapular muscles play a major role in healthy upper-extremity movements.

While these results are promising, the shoulder model has its limitations. First, we presented comparisons for tasks performed by a single healthy subject. The inherent variability amongst individuals and particularly patients with varying pathologies calls for much more comprehensive testing. Second, scaling the model and particularly thoracic muscle paths was an arduous and time-consuming task. In some muscles, such as the rhomboids, the range of motion of the scapula resulted in fibers either being too short or too long to generate sufficient active force. In these cases, we had to increase optimal fiber length and to reduce tendon slack length for the muscles to generate force over the full range of motion. There is considerable work to be done to automate the scaling of the scapulothoracic joint and associated muscle paths and parameters. Third, the glenohumeral joint was modeled as a ball-and-socket joint, thereby ensuring the stability of the joint and reducing the need for rotator-cuff muscles. Nonetheless, rotator-cuff muscle forces required for joint stability (Cain et al., 1987; Lippitt and Matsen, 1993) are not expected to contribute significantly to the total muscle work reported in this study because: i) their contribution to reaction forces increases, but reactions do not perform work, and ii) their elevation/abduction moment-arms are small (Yanagawa et al., 2008). We recognize that stability of the glenohumeral joint remains necessary to accurately estimate rotator-cuff forces and glenohumeral reaction forces (Ameln et al., 2019).

## Conclusions

Diagnosing, treating and augmenting human performance requires deep understanding of the function of muscular and skeletal structures that produce healthy and pathological movements. The activity and work done by individual muscles provides insight into the actions of muscles. Since the pioneering model and analysis of the shoulder mechanism (van der Helm, 1994b), there has been little reported about shoulder muscle forces and work to move the scapula and the arm. We developed a model that includes both the musculature and degrees-of-freedom of the human shoulder, which we combined with experimental data to compute the work done by large thoracoscapular muscles. We showed that of these muscles, the trapezius and serratus anterior muscles combine to do the majority of the work of upward rotating the scapula and elevating the arm.

The shoulder model and simulation environment (OpenSim) are provided freely from SimTK.org (https://simtk.org/projects/thoracoscapular). The model runs natively in OpenSim without third party dependencies. Clinicians, researchers and students can probe the model for muscle and joint reaction forces from the analysis of subject and patient motion capture data as we have demonstrated. The capability of running the model in a purely forward dynamics simulation also makes the model suitable to ask “what if?” questions. For example, in the case that serratus anterior is weakened, can external bracing prevent winging? If so, why might bracing outcomes vary widely (e.g., Vastamäki et al., 2015)? Or, can the model elevate the arm if serratus anterior is incapacitated? If not, what rehabilitation strategy or assistive device can support the role of serratus anterior to enable arm elevation? These and other questions can now be explored with our model.

## Data Availability Statement

The model and simulation environment (OpenSim) are freely available, deployable, and modifiable for any research or commercial use without restrictions from SimTK.org at https://simtk.org/projects/thoracoscapular and https://simtk.org/projects/opensim, respectively. The shoulder model does not require additional third party environments or software. Scripts for batch processing the analyses in this study are provided as MATLAB files.

## Ethics Statement

This study was carried out in accordance with the recommendations of the Ethics Committee of the Polytechnic Institute of Setúbal with written informed consent from all subjects. All subjects gave written informed consent in accordance with the Declaration of Helsinki. The protocol was approved by the Ethics Committee of the Polytechnic Institute of Setúbal.

## Author Contributions

AS and RM conceived of the shoulder model and study aims. AS supervised and performed data analysis and wrote first draft of the manuscript. MD extensively refined the muscle paths, performed the analyses, and generated the reported results. RM initiated the addition of thoracoscapular muscles and led the data collection effort. SD supported the study, contributed to study aims, and edited the manuscript.

### Conflict of Interest

The authors declare that the research was conducted in the absence of any commercial or financial relationships that could be construed as a potential conflict of interest.
